# What is the relationship between people with dementia and their caregiver’s illness perceptions post-diagnosis and the impact on help-seeking behaviour? A systematic review

**DOI:** 10.1177/1471301221997291

**Published:** 2021-03-31

**Authors:** Jane E Gregg, Jane Simpson, Ramin Nilforooshan, Guillermo Perez-Algorta

**Affiliations:** Department of Health Research, 4396 Lancaster University, Lanchester, UK; Department of Health Research, 4396 Lancaster University, Lancaster, UK; Research & Development Department, 9490 Surrey and Borders Partnership NHS Foundation Trust, Chertsey, UK; Department of Health Research, 4396 Lancaster University, Lancaster, UK

**Keywords:** dementia, Alzheimer’s, help seeking, illness perceptions, illness representations

## Abstract

**Background:** As the number of people with dementia increases, more families will be affected by the daily challenges of providing effective support, given its current incurable status. Once individuals are diagnosed with dementia, the earlier they access support, the more effective the outcome. However, once people receive a diagnosis, how they make sense of their dementia can impact on their help-seeking intentions. Exploring the illness beliefs of people with dementia and their caregivers and this relationship to help seeking may identify how best to facilitate early support.

**Aims:** To systematically obtain and critically review relevant studies on the relationship between illness perceptions and help seeking of people with dementia and their caregivers.

**Method:** A systematic search was conducted and included both quantitative and qualitative studies. The initial search was conducted in October 2018, with an adjacent search conducted in April 2020.

**Findings:** A total of 14 articles met the inclusion criteria. Conceptually, the studies examined the association of illness perceptions and help-seeking post-diagnosis and revealed that people living with dementia and their caregivers sought help when symptoms became severe. Components of illness perceptions revealed that lack of knowledge, cultural beliefs, complexity of the healthcare system, threat to independence and acceptance were identified as major factors for delaying help seeking.

**Conclusion:** Although research interest in the area of illness perceptions and their impact on help seeking for dementia is increasing, further work is needed to understand this area, particularly regarding the influence of the relationship between the person with dementia and their caregiver.

## Introduction

Currently, there is no cure for dementia; however, an early diagnosis can bring significant social, personal and economic benefits, which can impact on improving the quality of life for people living with the condition (Perry-Young et al., 2018). Further, seeking out help for interventions, either pharmacological or psychological, at the earlier stages of the disease can be related to milder impairment. For example, a study by Tang et al. (2016) revealed that individuals with dementia who sought help later had worse depressive symptoms and neurological functioning than people who had received treatment earlier. Moreover, a study by Moon et al. (2017) revealed that caregivers reported that the person with dementia was significantly less involved in decision-making for daily support and valued social contact less than their caregiver.

Thus, the notion of accessing treatments for dementia care earlier rather than later is important and is at the core of living well with dementia strategies from governments worldwide (e.g. UK Prime Minister’s Challenge on Dementia, 2012). Good post-diagnostic support for people living with dementia and their caregivers can facilitate a better understanding of their condition, as people living with early-stage dementia can potentially plan for their future while still able to do so, enhancing their quality of life (Devoy & Simpson, 2016). However, once people are diagnosed, during the early stages of dementia, they and their caregivers are often reluctant to seek help, as dementia still attracts a level of shame and stigma due to its links with diminished capacity, poorer mental health and loss of independence (Herrmann et al., 2018).

To ameliorate this situation, this review summarises information about the association of illness perceptions with help-seeking intentions, as to provide a framework to understand the components that form an individual’s illness perceptions once diagnosed with dementia. The role of illness perceptions has long been acknowledged as an important part in responding to symptom recognition and self-management of diseases or conditions generally (Hagger & Orbell, 2010) and in relation to dementia specifically (e.g. Roberts et al., 2014). There have been several proposed definitions of illness perceptions, comprising different models that include the cognitive and emotional components of a person’s representation of their illness. For a more detailed presentation, see Petersen et al. (2011). These processes are important as they can influence an individual’s coping strategies once diagnosed, involving risk perception and psychological well-being.

The Self-Regulatory Model (Leventhal & Meyer, 1980) is a useful model for understanding the coping processes and beliefs relating to an illness. This model explains how individuals perceive their illness via cognitive representations, such as identifying with the disease, cause/control, consequences, coherence and the emotional response to the illness (Shinan-Altman & Werner, 2019). Therefore, illness perceptions and their relationship to help seeking are important determinants of the individual’s management of their illness. Sometimes, these lay representations will coincide with scientific orthodoxy and sometimes they will be at odds with more accepted beliefs around the condition. Thus, understanding how people make sense of dementia and its implications is an important issue when working with individuals as they come to terms with their dementia diagnosis (Harman & Clare, 2006).

A recent systematic review of help seeking for dementia (Werner et al., 2014) examined non-professional and professional sources of help seeking, with results showing a preference for seeking help from close family members and friends followed by primary healthcare services. However, this review did not explore the mechanisms implicated in the process of help seeking, such as illness perceptions.

While research in the area of help seeking for dementia has been increasing, to the best of our knowledge, there has not been a review exploring how the person with dementia and their caregiver’s illness perceptions impact on help-seeking intentions once diagnosed with dementia. Therefore, a clearer understanding of how people’s illness perceptions and the relationship to help seeking once diagnosed may provide insight into an individual’s attempt to manage the illness. Thus, the aim of this review was to provide a preliminary evaluation of the available literature (qualitative and quantitative) on the relationship between illness perceptions with help seeking with people diagnosed with dementia and their caregivers.

The specific review questions are as follows:

1. How do illness perceptions impact on the intention to seek help after a diagnosis of dementia?
2. How does a caregiver’s illness perceptions impact on their intention to seek help for the person with dementia and for themselves?

## Methods

### Search strategy and selection of studies

The methodology applied for this review was based on the Evidence for Policy and Practice Information and Co-ordinating Centre guidelines (EPPI-Centre; Oliver et al., 2005), which was designed for wide-ranging research questions including both quantitative and qualitative evidence (Clement et al., 2014). The EPPI-Centre incorporates an initial scoping and mapping exercise to specify and prioritise any relevant studies. After conducting a scoping review, this revealed two main types of literature: qualitative and quantitative.

**Figure 1.**
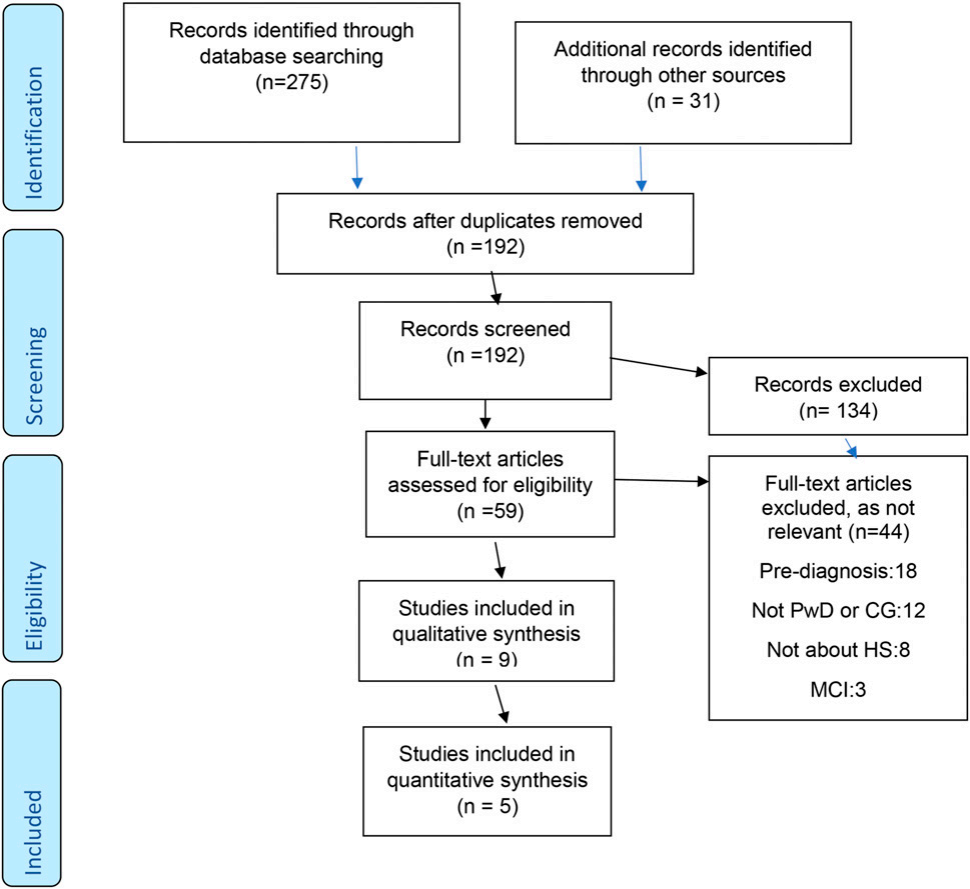
Preferred Reporting Items for Systematic Reviews and Meta-Analyses (PRISMA) flow diagram. MCI: mild cognitive impairment; HS: help seeking, CG: caregiver, PwD: people with dementia.

In line with the EPPI-Centre method, a parallel review was conducted for the quantitative and qualitative studies, with findings from both reviews brought together in juxtaposition in a meta-synthesis. The Preferred Reporting Items for Systematic Reviews and Meta-Analyses (PRISMA) checklist guidelines for the conduct of the findings were applied (see Figure 1). As this literature review incorporated a broad subject area, a search of general databases was conducted utilising Cochrane Central Register of Controlled Trials, Cochrane Dementia and Cognitive Improvement Group, ALOIS and Centre for Reviews and Dissemination; however, this search did not identify any relevant studies; thereafter, more specific health-related databases were searched. These were Cumulative Index to Nursing and Allied Health Literature, PsycINFO, MEDLINE and PubMed. Furthermore, each individual database was searched with relevant subject headings from February 2018 to August 2018 and revised in October 2018. An adjacent search was conducted in April 2020. Search terms were identified in collaboration with a specialist librarian. The search terms used were dementia or ‘vascular dementia’ or ‘Alzheimer’s’ or ‘Lewy body’ or ‘frontotemporal’ and were applied as MeSH terms which produced >94,000 hits. Thereafter, the search was modified with search terms aimed to represent the primary concepts of ‘dementia’, ‘help seeking’ and ‘illness perceptions’. Keywords entered were ‘Illness perceptions and Alzheimer’s and help seeking’ ‘Illness representations or help seeking’ ‘dementia and caregivers or help seeking or illness perceptions’. Adjacent search terms were ‘Identity’ or ‘control’ or ‘cause’ or ‘timeline’ or ‘consequences’ or ‘emotion’ or ‘coherence’ and ‘dementia’ and ‘help seeking’ The search process was also enhanced by manual searching of reference lists. Experts in the field were also contacted for any ongoing/or unpublished studies. Additionally, grey literature was searched on electronic databases (OpenGrey, BASE). Once articles were identified through this database search, the main reviewer (JG) screened titles and abstracts to assess eligibility.

### Inclusion criteria

- Studies that identified key terms in title, abstract or MeSH headings were retained.
- Inclusion criteria were studies that explored relationships between illness perceptions and help-seeking intentions/behaviours for people diagnosed with dementia and their caregivers and receiving informal care in the community.
- The term ‘perception’ did not have to be applied, as studies looking at these associations can use other terms such as illness ‘representations’, ‘cognitions’ or ‘beliefs’
- Articles published in peer review journals and written in English.

### Quality assessment

Before the quality assessment was conducted, an agreed standardisation of 80% level of agreement was considered acceptable between two reviewers (JG and RN). The second reviewer, RN, is a consultant psychiatrist specialising in dementia. The two reviewers independently assessed the qualitative studies applying the Critical Appraisal Skills Programme (CASP, 2018) checklist assessment tool. The main reviewer (JG) assessed all qualitative studies, with the second reviewer (RN) independently assessing a random sample (*n* = 5) of paper’s and clarified inconsistencies with the main reviewer for rigour and suitability for the review. The CASP checklist was designed as a tool within educational workshop settings; thus, a scoring system is not recommended; moreover, this format has been deemed appropriate for assessing qualitative studies (CASP, 2018).

For the quantitative studies, methodological quality was also assessed (JG and RN) by using the cross-sectional survey checklist (Centre for Evidence Based Management, 2014, adapted from Crombie, 1996). The main reviewer (JG) and second reviewer (RN) independently assessed studies using checklist criteria and resolved discrepancies through mutual discussions. Figure 1 details the final selection of studies.

### Data extraction strategy

Data from all studies were extracted by the main reviewer (JG) using a data extraction tool adapted from Egan et al. (2003). Standard study characteristics were extracted, plus details of study design, outcome measures and main findings. Using this tool aided in the collating of data from selected articles and helped identify differences and similarities in terms of key findings and methodology.

### Data synthesis

Findings were synthesised applying standard methods for narrative synthesis (Popay et al., 2006). Narrative synthesis was utilised as there was a substantial clinical and methodological heterogeneity between all studies. Moreover, a meta-analysis was not deemed appropriate as heterogeneity was considerable across selected studies in respect of primary outcomes, types of dementia and cultural differences. Therefore, the analysis incorporated a compare and contrast approach by conducting a comprehensive juxtaposition of review findings across all identified studies (Popay et al., 2006). Additionally, a tabular presentation of the characteristics of the identified studies was included to support the narrative and to aid in identifying patterns across the data (see Tables A1 and A2 in Appendix A).

### Data analysis

Qualitative studies were analysed by the main reviewer (JG), adopting a thematic analysis (Braun & Clarke, 2006). Thematic analysis provides a tool to analyse and identify themes unrestricted from any theoretical undertakings and has been applied successfully when synthesising various data sets, enabling flexibility within various theoretical paradigms (Bunn et al., 2012). Illness perception dimensions were noted by identifying recurring and prominent themes and allowed for categories to emerge from the data. This process allowed for grouping and regrouping of relevant data associated with illness perceptions. Thereafter, data were revised to identify interrelated themes and subthemes across and within the data set to form a final set of emergent themes (Clarke & Braun, 2017).

Quantitative studies were also analysed by the main reviewer. For quantitative studies, values representing the association between perceptions/cognitive processes and help seeking were extracted, and illness perceptions grouped into identity, cure/control, cause, consequences, coherence and emotional representations (Leventhal & Nerenz, 1985). The relationship between illness perception dimensions and reported outcomes was based on an examination of the author’s interpretations of data-specific sets that supported the relationship and its direction (Clement et al., 2014).

## Findings

### Included studies

As noted in Figure 1, a search of databases was completed and yielded 275 references. Thereafter, 31 additional references were identified via other sources. After removal of duplicates and studies that clearly did not meet the inclusion criteria, 192 full text records were retrieved. Of these 192 records, a further 134 were excluded at this point as not being relevant, leaving 59 full text references to be assessed further for eligibility. Of these remaining records, 44 studies were excluded as they did not meet all the inclusion criteria. Therefore, 14 studies were eligible to be included in this review. Nine studies were qualitative and five were quantitative. See Figure 1 for PRISMA flow chart diagram of search process.

### Study and participant characteristics

The selected studies were from various backgrounds (psychology, psychiatry, public health, mental health nursing and dementia) and included articles from various countries. In summary, seven studies were US based, two UK based and two Australia based, and there was one study each from China, Hong Kong, Vietnam and Europe (eight European countries in total, including the UK). In relation to study settings, community-based scenarios were day-care units, dementia clinics, support groups and a roadshow. For participants recruited into the studies, 11 studies involved caregivers, with only three investigating people with dementia as well as their caregivers. Regarding the approach applied for data collection for the qualitative studies, four applied semi-structured interviews (Au et al., 2013; Haralambous et al., 2014; Mukadam et al., 2015; Peterson et al., 2016), two focus groups (Braun et al., 1996; Stephan et al., 2018), one an unstructured interview, (Browne et al., 2007), one was descriptive (Braun & Browne, 1998) and one employed a roadshow/discussion format (Parveen et al., 2017). For the five quantitative studies, four applied a survey design (Hinton et al., 2006; Phillipson et al., 2013; Smyth & Milidonis, 1999; Valle et al., 2004) with no follow up and one was a longitudinal survey (Cox, 1999) with two follow up evaluations over a 12-month period.

### Quality appraisal: Qualitative studies (*n* = 9)

The CASP (2018) checklist tool assessed for quality regarding justification for methods used, data source collection and analysis, and all studies were considered appropriate. However, most studies (*n* = 6) did not either report informed consent procedure or confidentiality processes. Moreover, all included studies did not adequately describe the relationship between the researcher and participants, with no reflection on any potential influence regarding collecting and analysing data. Only two studies (Haralambous et al., 2014; Parveen et al., 2017) applied a theoretical framework. Table A3 reports on the methodological issues for all included qualitative studies (see Appendix A).

### Quality appraisal: Quantitative studies (*n* = 5)

By utilising the cross-sectional survey checklist (Crombie, 1996), all studies applied measures that were reliable and valid. Furthermore, the samples utilised in all the studies were representative of the sample by reflecting similar characteristics among the population being researched. Additionally, only one study (Phillipson et al., 2013) reported confidence intervals for main results and only two studies (Cox, 1999; Phillipson et al., 2013) clarified the theoretical framework. Table A4 below outlines the methodological issues for the quantitative studies (see Appendix A).

### Emerging themes across all studies

The subthemes identified in the qualitative studies were also apparent in the quantitative studies. By comparing and contrasting findings across all studies, the five following themes were identified. Most frequent rated themes that emerged (>5) are presented in a tabular format in Table A5 (see Appendix A).

### Qualitative studies

The synthesis of qualitative studies produced findings relating to the illness perceptions of individuals and their identifying symptoms of dementia and the relationship of these to cultural beliefs and their impact on help seeking (Au et al., 2013; Braun & Browne, 1998; Mukadam et al., 2015; Parveen et al., 2017; Valle et al., 2004). One major theme related to cultural beliefs was the perceived consequence and the acceptance of duty of care from the caregivers (CGs). Seeking help can be construed as a weakness; thus, an unwillingness to seek help can be formed within an individual’s own perception of the consequences of caring for someone with dementia. This was noticeable in studies by Braun et al. (1996) and Braun and Browne (1998). They reported that Asian family hierarchal structures (i.e. duty to pay back to elders) influenced how people interrelated within their role as CGs. CGs’ own illness perceptions on the causes of dementia were seen as being attributed to normal ageing and within the family network went unnoticed. Moreover, perceptions of accepting a diagnosis of dementia were highlighted by Braun et al. (1996) and Braun and Browne (1998). These specific cultural beliefs can impact on seeking medical help, whereas CGs would only consider taking a person with dementia to a clinician if dementia symptoms were severe, in the belief that nothing could be done to cure them (Braun et al., 1996; Braun & Brown, 1998).

Secondly, findings revealed that an individual’s perception of the breadth of the concept of dementia could be an overwhelming experience for the person with dementia and the CG (Au et al., 2013; Browne et al., 2007; Haramlamblous et al., 2014). This lack of understanding about dementia could exacerbate the development of a coherent illness identity and could impact on an individual’s decision not to seek help. Furthermore, the quality of care experienced previously from health professionals could influence an individual’s tendency to seek help or not. If individuals had a negative experience, engagement became more difficult and professionals were rebuffed. Negative beliefs about residential and respite care were associated with non-use of these services (Haramlambous et al., 2014; Stephan et al., 2018).

Themes around an individual’s perception of the lack of controllability and coherence of dementia and the threat to independence in the context of living with dementia at home were apparent in articles by Stephan et al. (2018) and Peterson et al. (2016). In particular, the study by Stephan et al. (2018) reported that people’s attitudes and beliefs towards a diagnosis of dementia could impact on how they accepted the disease and then their subsequent use of formal care. These beliefs were reported as a major hindrance across all of the eight countries included in the article, suggesting that the person with dementia may lack insight into the symptoms associated with the condition and therefore lack awareness of their needs in respect of asking for help.

### Quantitative studies

Findings from the three association studies (Phillipson et al., 2013; Smyth & Milidonis, 1999; Valle et al., 2004) produced mixed conclusions. The study by Smyth and Mildonis (1999) reported a positive correlation among normative beliefs, derived from the CG’s own standards of caregiving and their relation to help seeking. CGs’ perceptions of the coherence of dementia and their own health were not significantly correlated to direct care tasks involving formal help providers, suggesting that help seeking was not influenced by caregivers’ own perception of cause and coherence of the severity of dementia symptoms. However, the quota of care tasks involving seeking help from formal helpers was marginally inversely correlated with Belief in Carer Independence (BCI) suggesting a small effect size. Despite the considerable variation of normative beliefs regarding the role of CGs and help seeking, there was a limited association between these beliefs and patterns of help seeking. For example, with BCI associated with carers’ feelings of being trapped, but preference for informal care and concern for family opinion was not. This suggests perceptions of the consequences of caring for someone with dementia can affect help seeking.

Valle et al. (2004) reported significant differences in caregiver experiences, with the strength of relationship between ethnic groups (Latino and Euro-American) and help seeking moderately strong (ethnic group factor explained 22% of variance of the dependent variable). Moreover, ethnicity was the only significant variable related to social network help seeking. Despite the strength of these associations between ethnicity and help seeking, the total model only accounted for 20% of variance in social network seeking scores which can be interpreted as a small effect size. Phillipson et al. (2013) used an expanded version of the Anderson Behavioural Model (ABM) (Anderson & Newman, 1973) to identify associated factors (health beliefs, perceived needs and social structures) with non-use of services. The ABM accounted for 42% of the variance in non-use of residential respite care and 67% for non-use of day care. This suggests that negative illness perceptions relating to controllability of the disease and emotional representations of CGs could result in negative outcomes for the person with dementia, as both were strongly associated with non-use. Overall, the model accounted for two-thirds of the variation of non-use of day care in relation to people’s perception of community services for dementia which can be interpreted as large effect size.

Cox (1999) and Hinton et al. (2006) investigated frequency distributions and patterns of use of services (i.e. professional help, support groups and day care). Cox’s study was the only longitudinal study over 12 months. Findings suggest that frequencies for both groups (African Americans [AA] and white caregivers) who sought support from services were similar (approximately 50% across both groups). The primary reason for seeking help was to obtain information on dementia, suggesting that attempts to create a coherent understanding of the disease facilitated help seeking. However, significantly more of the AA group requested day care than white CGs who enquired about support groups. Hinton’s article reported that a high percentage of CGs (80%) had sought help for at least one dementia symptom, with patterns of help seeking demonstrating that CGs reported disclosure of symptoms to the care recipient primary care provider. Furthermore, in Hinton’s study (2006), there were high levels of unmet needs for behavioural problems with >68% of CGs expressing a need for emotional support (counseling and information related to dementia). However, there was considerable variation in GG rates discussing neuropsychiatric symptoms with their family doctor, with 57% of GGs disclosing information about inappropriate elation, to 100% disclosing information about hallucinations. This suggests that CGs’ perceptions of the identity (symptom profile) of dementia can impact on what kind of help is sought.

## Discussion

This review sought to provide a narrative account of how the illness perceptions of people with dementia and their caregivers can impact on their tendency to seek help post-diagnosis. This review presents findings of 14 publications of which nine were qualitative and five were quantitative, with all studies exploring help seeking among people with a diagnosis of dementia living in the community. In contrast to previous reviews that examined the help-seeking intentions of people experiencing symptoms of dementia pre-diagnosis (Perry-Young et al., 2018; Werner et al., 2014), this review focused on help seeking once diagnosed. By synthesising the results from both qualitative and quantitative studies, a general consensus revealed that illness perceptions and the separate components that form these perceptions (symptoms/identity, cure/control, cause, consequences, coherence and emotional representations) were associated with barriers and facilitators to help seeking. These included strong cultural beliefs about symptoms of dementia, associating the disease as part of the ageing process. Also, inadequate knowledge and beliefs about dementia (coherence), and previous experiences of healthcare services (emotional representations and consequences), caused difficulty in identifying the symptoms of dementia and acceptance of a diagnosis (symptoms/cause/control).

Regarding quantitative studies, three of the five selected studies were association studies and, of these, two reported magnitude of effect sizes in relation to help-seeking intentions and an individual’s beliefs of dementia. Even though the sample of articles reviewed was small, findings were variable. Studies including frequencies and patterns of help seeking indicated that CGs were forthcoming in asking for help, specifically regarding information seeking. However, they also reported that the emotional burden of caring for someone with dementia could be a barrier for CGs regarding disclosing their own emotional distress for fear of being seen as unable to cope.

These findings were echoed in the qualitative synthesis process, where subthemes of emotional well-being and consequences were identified. These subthemes of emotional well-being, consequences and duty of care demonstrated how illness perceptions in relation to the stigma associated with caregiving may deter help seeking by various means. For example, that people were willing to dismiss the label of receiving formal care, as to avoid the public stigma this attracts, and the desire to avoid internalised feelings of embarrassment and shame (Corrigan, 2004).

Our findings show that individuals’ illness perceptions of dementia can contribute to a person’s help-seeking behaviour, with this review demonstrating the importance of cultural differences within approaches to help seeking, and how tailored interventions could be beneficial to individuals living away from their country of birth. However, it would also appear that people’s perceptions of their understanding of dementia, in relation to accessing health care, can impact on an individual’s tendency to seek out help. Also, there were reported instances of delays due to clinicians not identifying CGs’ issues of carer burden, and a lack of awareness, knowledge, and trust of dementia services.

A consensus from the studies reviewed is that people living with dementia only seek help when the symptoms start to become more severe. This suggests that an individual’s own perception about the severity of dementia can influence the time to seek out help. Barriers to seeking help are lack of knowledge and one’s own personal beliefs of dementia symptoms, suggesting that education about seeking help early on for dementia, rather than later, is much needed.

These findings seem to support previous literature on help seeking for dementia (i.e. Perry Young et al., 2018; Werner, 2003; Werner et al., 2014) and suggest that help seeking is a complex process that not only depends on the primary diagnosis but also how the individual makes sense of these changes. These illness perceptions are formed over time, suggesting the intention to seek help is part of a much longer process, as people come to terms with living with dementia (Perry-Young et al., 2018). As diagnostic procedures are becoming more available, it would seem advantageous for primary and community care services to offer interventions post-diagnosis to avoid further crises later (Burns, 2012).

## Limitations

A strength of this review is the inclusion of qualitative and quantitative studies, with a broad representative sample. However, we cannot disregard the possibility that some studies may have been missed due to publication bias (significant results more likely to be published). Furthermore, information was synthesised and reported in summary tables with no statistical techniques applied for examination of methodological issues. However, it should be noted that this review was intended to focus on methodological and conceptual developments and the impact on future clinical interventions and research, rather than an exhaustive review of the literature. Although inter-rater reliability was utilised for assessing the quality of studies, the data search, extraction and analysis were conducted by the first author, which may have influenced the identification of criteria used for initial inclusion of studies.

## Implications for practice

How people adapt and respond to a diagnosis of dementia is highly determinative of their future care, demonstrating that people’s perceptions of living and caring for someone with dementia can be an overwhelming experience. Therefore, it would be beneficial if a collaborative approach between health and social care sectors developed interventions after the initial diagnosis, to engage people who are hard to reach (Aldridge et al., 2019). Engaging people from the outset and supporting them as they adapt to living with dementia may encourage people to have a clearer understanding of the disease. Importantly, findings have shown that there is a delay in seeking help from community services once diagnosed with dementia due to a lack of trust in dementia services and, as people can be referred back to primary care after a diagnosis, a breakdown in communication can occur. After an initial diagnosis, there is little clinicians can offer under community mental health services, suggesting a need for more support at this time point by incorporating a more joined up process at the early stages of diagnosis and subsequent care from the family doctor. Receiving a diagnosis of dementia can be a daunting prospect for the person with dementia and their CG, with people displaying feelings of hopelessness. Thus, an individual’s own illness perceptions on dementia can influence their choices and contribute to their help-seeking behaviour.

## Conclusion

This review set out to explore and understand how people with dementia and their caregivers seek help after a diagnosis of dementia in relation to their own illness perceptions. In summary, studies in the area of help seeking and dementia have been increasing over the past two decades, indicating a greater interest in an understanding of this concept; however, there remains a gap in the current literature. This review highlighted how the components of illness perceptions and their association with cultural beliefs, lack of knowledge, stigma, acceptance of the condition and experience of services for dementia care can all play part in effecting how people seek out help. However, these processes are formed over time and as people balance their own beliefs and cognitions with the acceptance of living with dementia, the need to seek out help is a long process, rather than occurring at one single time point (Perry-Young et al., 2018). Furthermore, given that stigma can impact on help seeking (Clement et al., 2014), developing strategies to reduce stigma-related issues need to be addressed. A number of interventions do exist, aimed at effecting, for example, societal and individual change (Link & Phelan, 2006). Moreover, only three studies explored the person with dementia’s illness perceptions with help seeking, with the relationship between the person with dementia and the CG not considered, suggesting that more research is needed in this area. Therefore, it would seem advantageous for future research to develop interventions addressing the factors highlighted in this review, in respect of the long-term effects of living with dementia in the community.

## Appendix A

**Table A1.** Study characteristics of qualitative studies.

Reference	Objective	Design	Sample	Setting	Analysis	Illness perceptions/themes	Outcomes
Braun et al. (1996)	To explore perceptions of Vietnamese immigrants in the USA, regarding caregiving and help seeking of a PwD	Focus groups	Four groups. Mean number in groups = 11.5: Men (mean age 65.2) women, (mean age = 55.6) youth (mean age = 23.8) and mixed groups of CG of person with dementia (mean age = 54.0)	Community-Vietnam	Not mentioned	Identity, consequences, control: Duty of care – cultural beliefs	Results reported importance of hierarchy family structures in the Vietnamese population, with a low priority of dealing with dementia when facing problems associated with caring, and a willingness to access services
Braun and Browne (1998)	Presents information on how cultural values and practices affect perception of dementia, caregiving and help seeking	Descriptive	APIs	Community-USA	Descriptive	Identity, control, emotional representations: Duty of care – cultural beliefs/stigma	Cultural beliefs can affect individuals asking for help, this can be seen as a weakness. Family norms dictate the beliefs around responsibility to care for person with dementia
			Age not reported				
Browne et al. (2007)	To gain an understanding of help seeking process of older husbands CG of wives with dementia	Unstructured interviews	9 CG of persons with dementia mean age = 79 years, range 65–87 years	Community-USA	Grounded theory	Consequences, cure/control: Complexity of system – negative and positive experiences	Main findings were that attitudes, values, and experiences influenced choices made, especially the influence of negative previous experiences with care providers
Mukadam et al. (2015)	To explore link between attitudes to help seeking for dementia in ME people and the indigenous population	Semi-structured interviews	18 CGs of person with dementia. Mean age = 57 years	Community-UK	Thematic analysis	Identity, cure/control: Duty of care – cultural beliefs/stigma	All carers seemed to identify early symptoms of dementia, however barriers to early help seeking in the ME population was that a dementia diagnosis was of no use, and that it was a family’s duty to care for person with dementia
Au et al. (2013)	To explore coping and help seeking behaviour among Hong Kong CG of PwD	Semi-structured interview	11 CG of persons with dementia. Age range = 43–83 years	Community- Hong Kong	Grounded theory	Emotional representations: Complexity of system – experiences and response from HP	Internal regulation, forbearance and family obligations are linked to not seeking help earlier. Chinese CG may be hesitant about disclosing information and seeking help, as were found to approach family for help rather than HP.
Haralambous et al. (2014)	To determine barriers and enablers to accessing dementia services among older Asian PwD in Melbourne	Semi-structured interview/cultural exchange model	12 CG of person with dementia mean age of Chinese CG = 54 years	Community-Australia	Cultural exchange odel	Identity, cure/control: Complexity of system – negative positive experiences	Barriers to accessing services included complexity of health system, language barriers and lack of knowledge about dementia
			Mean age of Vietnamese CG = 62 years				
Peterson et al. (2016)	To understand complex determinants that lead CG of dementia need for education and assess barriers to seeking help	Semi-structured interview	27 persons with dementia and CG. Mean age of CG = 58 years. Mean age of PwD = 79.8 years	Community-USA	Content analysis	Identity, cure/control/consequences: Lack of knowledge – symptoms and cause	Barriers to seeking help were linked to knowledge gaps about dementia rather than reluctance to assume CG role. More public education for CGs for person with dementia is needed
Parveen et al. (2017)	To explore perceptions of dementia and use of services among various ethnic community	Roadshows/discussion groups	175 persons with dementia, carers and community members. Age not reported	Community-UK	Thematic and framework analysis	Identity, cause, emotional representations: Threat to independence – hindrance or help – cultural beliefs	Seeking help from services seen as a hindrance, linked to a lack of awareness about dementia and cultural barriers such as religious beliefs and language
		SRM					
Stephan et al. (2018)	To explore barriers and facilitators to access formal dementia care	Focus groups	147 persons with dementia and CG. Mean age of person with dementia = 76 years. Mean age of CG = 63 years	Community-8 European countries	Content analysis	Identity, cure/control/consequences: Lack of knowledge – symptoms and cause	Formal care be a threat to an individual’s independence by the PwD. Health professionals seen as key contact

CG: caregiver; API: Asian Pacific Islander; ME: minority ethnic; HP: health professional; SRM: Self-Regulatory Model.

**Table A2.** Study characteristics of quantitative studies.

Reference	Objective	Design/measures	Sample	Setting	Analysis	Illness perceptions/themes	Outcomes
Cox (1999)	Exploring experiences of AAs and white CG seeking assistance for person with dementia	Longitudinal/survey/Anderson Behavioural Model	300 CGs of person with dementia	Community-USA	Chi-square/t-test/contingency analysis	Cure/control, consequences: Acceptance of diagnosis/emotional well-being	Both groups showed symptoms of clinical depression. Primary reason for seeking help was to obtain information on dementia. With significantly more AA calling for home help (<.001) or day care (*p* < .001), while more white CG (*p* < .05) called about support groups
		ADL, IADL CES-D	150 white CG, mean age = 57 years, 150 AA mean age = 54 years				
Smyth and Milidonis (1999)	Study the relationship between exploration of service use, normative beliefs and help seeking	Survey/psychological scales: CATSI, COO and PIC	120 CG and person with dementia, mean age of CG = 67 years	Community-USA	ANOVA/correlation	Consequences/emotional representations: Acceptance of diagnosis/emotional well-being/consequences (captivity)	3 subscales significantly correlated: BCI and CFO (*r* = .32, *p* < .001) BCI and PIC (*r* = .61, *p* < .001) CFO and PIC (r = .22, *p* < .01). Normative beliefs regarding accessing help were significantly positively associated with CG physical and mental health
Hinton et al. (2006)	To examine dementia neuropsychiatric symptoms severity and help-seeking patterns	Survey/neuropsychological scales NPI, CES-D and ADL	38 CGs of persons with dementia. Mean age = 70 years	Community-USA	Chi-square	Identity: Complexity of the system – responses from HP, negative & positive experiences	CG perceived unmet needs for professional help in relation to specific NPI symptoms (75% disinhibition, 66.7% delusions). 80% of CG had seeked help for at least one neuropsychiatric symptom
Valle et al. (2004)	Ethnic differences in social network help-seeking strategies	Surveypsychological scales: ASSIS, MBC and WOC-R	89 persons with dementia and CGs. Euro-Americans *n* = 50, mean age of CG = 69 years. Latino *n* = 39, mean age of CG = 57 years	Community-USA	Chi-Square/t-test/multiple regression	Cure/control, emotional representations: Duty of care – cultural beliefs	Accounting for 21% variance of social network help seeking, the relationship between ethnicity and help seeking was moderately strong b = −3, *p* = .04
Phillipson et al. (2013)	Why carers of PwD do not utilise out of home services	Survey/psychological scales: ZBI, ADL and CES-D	152 CGs of persons with dementia. Mean age of CG = 66.36 years	Community-Australia	Univariate analysis/chi-square t-test	Cure/control, emotional representations: Lack of knowledge – symptoms and cause	Beliefs that service use would result in negative outcomes for persons with dementia were strongly associated with non-use of day care (OR 13.11 95% CI (3.75, 45.89) and respite care (OR 6.13 95% CI (2.02, 18.70). ABM accounted for 67-42% variance in non-use of day centres
		ABM					

CATSI: caregiver for attitudes toward services inventory; BCI: Belief in Caregiver Independence; PIC: preference for informal care; CFO: concern for family opinion (Collins et al., 1991); COOs: concern for the opinion of others; ASSIS: Arizona Social Support Interview Schedule (Barrio, 2000) MBC: Memory and Behaviour checklist WOC-R: Ways of Coping Revised (Vitaliano et al., 1985); ZBI: Zarit Burden Inventory (Zarit et al., 1998), ADL: activities of daily living; IADL: independent activities of daily living; (Zarit & Zarit, 1987) NPI: Neuropsychiatric Inventory Scale (Cummings et al., 1994); CES-D: Centre for Epidemiological Studies Depression Scale (Radloff, 1977) PwD: person with dementia, CG: caregiver, AA: African American, ABM: Anderson Behavioural Model (Anderson & Newman, 1973), SRM: Self-Regulatory Model (Leventhal & Meyer, 1980).

**Table A3.** Methodology issues for qualitative studies.

Reference	Design	Methodology issues
Braun et al. (1996)	Focus groups – audio taped	No mention of informed consent
Browne et al. (2007)	Face-to-face unstructured interviews – audio taped	Convenience sampling. Participants recruited through support groups and personal contacts. Possibility for potential bias
Mukadam et al. (2015)	Face-to-face semi-structured interview – audio taped	Purposive sampling. Carers approached by clinician they knew. No mention of informed consent/confidentiality. Participants sent transcripts and invited to comment on accuracy
Au et al. (2013)	Face-to-face semi- structured interview – audio taped	Convenience sampling – no mention of researcher role in study
Haralambous et al. (2014)	Face to face semi-structured interview- audio taped	No mention of informed consent/confidentiality
Stephan et al. (2018)	Focus groups	Sampling procedure – not described adequately, participants contacted by gatekeepers: Support groups and known contact persons from other parts of the project
		No mention of informed consent/confidentiality

**Table A4.** Methodology issues for quantitative studies.

Reference	Model	Methodological issues
Cox (1999)	Andersen and Newman (1973)	No CI reported and limitations not reported
Smyth and Milidonis (1999)	Not stated	No CI reported and decision for sample size not reported
Hinton et al. (2006)	Not stated	No CI reported and small sample size (*n* = 38) in relation to epidemiological standards of Latino American people living with dementia
Valle et al. (2004)	Not stated	No CI reported and cultural issues not taken into consideration
Phillipson et al. (2013)	Andersen and Newman (1973)	Confounding factor of culture not reported

CI: confidence interval.

**Table A5.** Identified themes in relation to illness perceptions.

Illness perception	Theme	Subtheme
Identity/cure/control	Duty of care	Cultural beliefs/stigma
Cure/control/emotional representations/	Threat to independence	Hindrance or help
Consequences/emotional representations/coherence	Complexity of system	Response from health professional. Negative and positive experiences
Coherence/identity/cause	Lack of knowledge	Symptoms and cause
Identity/cure/control/emotional representations	Acceptance of diagnosis	Emotional well-being/consequences
